# Invasion success of a Lessepsian symbiont-bearing foraminifera linked to high dispersal ability, preadaptation and suppression of sexual reproduction

**DOI:** 10.1038/s41598-023-39652-y

**Published:** 2023-08-03

**Authors:** Débora S. Raposo, Rebecca A. Zufall, Antonio Caruso, Danna Titelboim, Sigal Abramovich, Christiane Hassenrück, Michal Kucera, Raphaël Morard

**Affiliations:** 1 Center for Marine Environmental Sciences, MARUM, Universität Bremen, Bremen, Germany; 2 Department of Biology and Biochemistry, University of Houston, Houston, USA; 3 Dipartimento di Scienze e Tecnologie Biologiche Chimiche e Farmaceutiche, Università degli Studi di Palermo, Palermo, Italy; 4 Department of Earth Sciences, University of Oxford, Oxford, UK; 5 Department of Earth and Environmental Sciences, Ben Gurion University of the Negev, Beer Sheva, Israel; 6 Department of Biological Oceanography, Leibniz Institute for Baltic Sea Research Warnemünde (IOW), Rostock, Warnemünde, Germany

**Keywords:** Molecular biology, Invasive species, Genetics, Eukaryote, Genotype, Haplotypes, Population genetics, Sequencing

## Abstract

Among the most successful Lessepsian invaders is the symbiont-bearing benthic foraminifera *Amphistegina lobifera.* In its newly conquered habitat, this prolific calcifier and ecosystem engineer is exposed to environmental conditions that exceed the range of its native habitat. To disentangle which processes facilitated the invasion success of *A. lobifera* into the Mediterranean Sea we analyzed a ~ 1400 bp sequence fragment covering the SSU and ITS gene markers to compare the populations from its native regions and along the invasion gradient. The genetic variability was studied at four levels: intra-genomic, population, regional and geographical. We observed that the invasion is not associated with genetic differentiation, but the invasive populations show a distinct suppression of intra-genomic variability among the multiple copies of the rRNA gene. A reduced genetic diversity compared to the Indopacific is observed already in the Red Sea populations and their high dispersal potential into the Mediterranean appears consistent with a bridgehead effect resulting from the postglacial expansion from the Indian Ocean into the Red Sea. We conclude that the genetic structure of the invasive populations reflects two processes: high dispersal ability of the Red Sea source population pre-adapted to Mediterranean conditions and a likely suppression of sexual reproduction in the invader. This discovery provides a new perspective on the cost of invasion in marine protists: The success of the invasive *A. lobifera* in the Mediterranean Sea comes at the cost of abandonment of sexual reproduction.

## Introduction

Biological invasions driven by climate change are currently profoundly modifying ecological landscapes^1,2^. Unlike normal range extensions, where species are largely tracking their climatic envelope, invasive species conquer entirely new spaces, with a higher probability of facing climatic (seasonality), physical (light), chemical (salinity) or biotic (microbiome and interactome) conditions that exceed the range they experienced within their native habitat. In this context, it is important to understand how a given species can become a successful invader. The challenge of exposure to foreign conditions in the newly conquered space could be counteracted by adaptations. In this scenario, successful invaders can be expected to display a high adaptive potential^3,4^. Alternatively, the native population could already possess the key adaptations, for example as a result of its evolutionary history^5,6^, past migration events^7^, or ecological filtering^8^.

A remarkable biological invasion phenomenon known as the Lessepsian invasion has taken place in the Mediterranean Sea since 1869. The opening of the Suez Canal in that year ignited a dramatic and largely uni-directional migration of Indo-Pacific marine species into the Mediterranean. So far, over 600 invasive marine species have been reported in the eastern Mediterranean^9,10^, with more new invaders appearing as the ongoing warming makes the Levantine Basin more tropics-like^11^. Among the particularly successful invaders are symbiont-bearing larger benthic foraminifera (LBF). LBFs inhabit shallow coastal waters, where they commonly live attached to algae or hard substrate^12^. Foraminifera have limited capacity for active movement during life, but adult specimens can be passively suspended and transported by currents^13^ and the passive mobility of the minute juveniles, propagules, or flagellated gametes produced by sexual reproduction is likely even larger^14,15^. In addition, foraminiferal dispersal mediated by travel in the digestive system of fish was documented in the Mediterranean^14,14^. This combination of multiple mechanisms of dispersal results in broad species ranges and lack of regional population differentiation^17^.

The most successful Lessepsian invader LBF is the diatom-bearing *Amphistegina lobifera* Larsen 1976, whose prolifically calcifying invasive populations modify the sediment substrate and displace native species^18,19^. After establishing itself in the Levantine Basin, its invasion towards the west accelerated in the last few decades^18,20–26^ and the species now expanded its range to Sicily^27^*.* The invasion appears to be sourced entirely from within the western Indian Ocean genotype Ia of the species^17^, but it remains unknown how the genetic diversity of the invading populations is structured along the invasion gradient.

Theoretically, an invading population could show a reduced genetic diversity compared to the source population because only a fraction of the source population participated in the invasion (founder effect^28–30^) or because the source population is highly structured and only one subpopulation possessed adaptations allowing it to invade^31–33^. The combination of a founder effect and exposure to the new environment could also lead to rapid emergence of new variation in the invasive population (e.g., as observed in a Lessepsian cornetfish^34^). In both cases, the reduced variability and increased divergence should show a gradient along the invasion progression^35,36^, with most severe effects visible at the invasion front. Alternatively, the source population could have already possessed the necessary adaptations to the new environment and in the presence of a large dispersal potential^17^, the opening of the Suez Canal could have merely removed a physical barrier after which the invader rapidly fills the free space without genetic differentiation.

Here we investigated the population structure of the invasive *A. lobifera* between the source population and populations representing different stages of the invasion. The analysis is based on a ~ 1400 bp long sequence fragment of the rRNA gene complex covering the end of the SSU coding region and the adjacent internal transcribed spacer (ITS) region. In foraminifera, the SSU rRNA gene contains fast-evolving variable regions, which provide a resolution within species^37–40^ and the ITS rRNA gene region provides an even higher resolution given its higher mutation rate^41^. Therefore, this marker should allow us to detect genetic differentiation (or a lack thereof) among the invasive populations of *A. lobifera* and thus constrain to what degree the invasion success of the species is due to novel adaptive change or preexisting adaptations.

## Materials and methods

**Figure 1:**
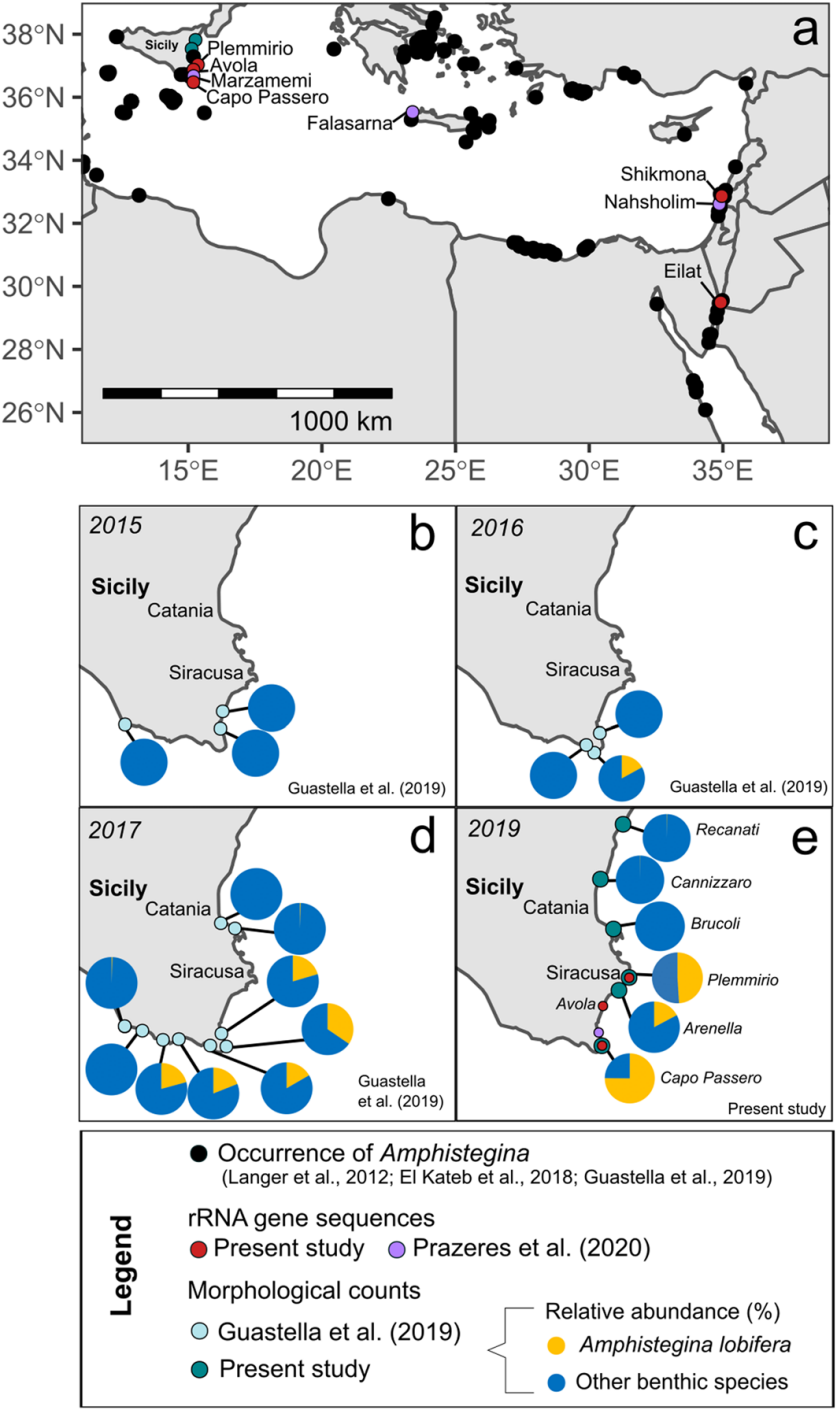
Occurrence of invasive *Amphistegina* in the Mediterranean Sea and sites sampled for genetic analysis (a, and e for zoom in the Sicilian sites) and progress of invasion in Sicily from 2015 until the present study assessment in 2019 (**b**)–(**e**). The map was created with the World Map Data from Natural Earth R package “*rnaturalearth*” version 0.1.0 available at https://CRAN.R-project.org/package=rnaturalearth.

To characterize the genetic variability of *Amphistegina lobifera* along the invasion gradient, we sampled populations of the species in Sicily, where the most recent invasion front has been identified by Guastella et al.^27^, and in Israel where populations representative of the source population (Red Sea) and pioneer invaders (Eastern Mediterranean Sea) could be collected (Fig. 1). During the sampling in Sicily in September 2019, we first carried out an exploratory survey to identify the position of the invasion front compared the observations of Guastella et al.^27^ that were done between 2015 and 2017. We focused our effort on the eastern coast of Sicily, where the invasion front was located by Guastella et al.^27^ between Capo Passero to the South and Brucoli to the North. We revisited these two locations and sampled two additional locations in between (Arenella and Plemmirio) and two additional locations North of Brucoli (Cannizzaro and Recanati), assuming that the invasion front may have moved further North since 2017. At each location, pebbles and macroalgae were collected from the depth of 0.5–5 m by snorkeling. The collected substrates were brushed, and the recovered material was sieved at 63–500 µm and transferred in 0.5 L jars that were filled with ambient seawater. We then examined the samples under a stereomicroscope and assessed qualitatively the presence of *A. lobifera* in the samples.

We observed that the species was abundant at Capo Passero, Arenella and Plemmirio but absent or rare in the samples from Brucoli, Cannizzaro and Recanati (Fig. 1e). The collected samples for the exploratory phase were air-dried and transported to our laboratory in Bremen, Germany to quantitatively assess the progress of the invasion. The dried samples were weighed and split to obtain representative aliquots containing ~ 300 foraminifera per sample to determine the abundance of *A. lobifera* shells in the total assemblage (living + dead) and estimate its population density (individuals/g of sediment). The results are provided in Supplementary Table S1.

Following the exploratory survey, samples for genetic analyses were collected at Capo Passero and Plemmirio and at Avola located midway. The samples were collected as described above, but now living specimens of *A. lobifera* were isolated and individually transferred to micropaleontological slides, where they were air dried and stored at -20 °C (methods are detailed in Hallock et al.^42^; Schmidt et al.^25^; Stuhr et al.^43^). The isolated foraminifera were shipped on dry ice to our laboratory in Bremen, where they were stored at − 80 °C. The sampling in Israel was conducted in October 2019. We sampled at Shikmona, Haifa (Mediterranean Sea) and at Eilat, Gulf of Aqaba (Red Sea) where the presence of *A. lobifera* has already been documented^25,44,45^. The living specimens for genetic analyses were collected and isolated and transported as described above.

Between 10 and 24 specimens per site were selected for genetic analyses. We also analyzed nine specimens of *A. lobifera* collected at Okinawa, Japan (26.651819 N; 127.856243 E) in September 2015, that were present in our collection to serve as an outgroup. Each specimen was isolated in 50 µl of DOC buffer and the thick calcite shell was cracked with a sterilized crusher. Following the DOC protocol^46^, each specimen was incubated at 60 °C for 1 h followed by centrifugation at 10.000 rpm for 5 min and stored at 4 °C until further use.

Because we aimed at capturing a population dynamic process, we designed a protocol to access the most variable genomic region known for foraminifera, the Internal Transcribed Spacer (ITS)^41^ that is located between the ribosomal Small Sub-Unit (SSU) and the Large Sub-Unit (LSU). The genetic diversity of *A. lobifera* has been previously documented in the Indo-Pacific and the Mediterranean Sea based on a 600 bp fragment located at the end of the SSU (Prazeres et al. 2020). To make our results compatible with the existing data, we designed a protocol to amplify the same SSU fragment together with the entire ITS. We developed a semi-nested PCR protocol using the primers pairs S14F3 (5′-ACGCAMGTGTGAAACTTG-3′)—L5F (5′—TCGCCGTTACTAAGGRAATC—3′). and S14F1 (5′-AAGGGCACCACAAGAACGC-3′)—L5F^46^. The amplification was carried out using the GoTaq polymerase (Promega) with a PCR mix containing MilliQ water, 5× green buffer (final concentration: 1×), each primer (final concentration: 0.2 µmol/µl), MgCl_2_ (final concentration: 2.5 µmol/µl), dNTP mix (final concentration: 0.4 µmol/µl) and GoTaq polymerase (final concentration: 0.05 U/µl), and added DNA extract diluted 1:10 to reduce inhibition, within a final volume of 15 µl. The second PCR was carried out with the same mix but using 1 µl of the 1st PCR as the DNA template. The thermal cycling was as follows for both successive PCRs: initial denaturation at 95 °C for 2 min followed by 35 cycles of 30 s of denaturation at 95 °C, annealing for 30 s at 55 °C and extension at 72 °C for 45 s, followed by a final extension at 72 °C for 2 min.

The PCR product obtained was migrated on a 1.5% agarose gel and visually checked under UV light. The samples showing single bands were selected and purified using the QIAquick PCR purification kit (QIAGEN) following the manufacturer's instructions. Because of the presence of large intra-genomic variability among the multiple copies of the rRNA gene in foraminifera^40^, the purified PCR product was cloned using the TOPO® TA Cloning Kit (Invitrogen, USA). Amplicons were ligated to a pCR 2.1TOPO® vector, transformed into One Shot™ TOP10 chemically competent *Escherichia coli* cells, and grown overnight on LB-agar plates containing ampicillin (100 mg/ml). Eight to 16 clones per specimen were selected and placed in 1.5 ml tubes containing 30 µl of MilliQ water and a final PCR was performed and sent for Sanger sequencing with an external provider (LGC Genomics, Berlin). Due to the long fragment targeted (~ 1400 bp), each PCR product was sequenced from both ends using the primers S14F1 and L5F. The chromatograms were carefully checked and assembled, and the resulting sequences were deposited on NCBI under the accession numbers OP610171-OP610543. In addition to the new sequences, we also recovered all available *A. lobifera* sequences and associated metadata available on NCBI that were mostly produced by Prazeres et al.^17^ and Schmidt et al.^25^. We limited our query to sequences covering the entire ~ 600 bp fragment of the SSU, resulting in a total of 256 sequences. All newly generated sequences and publicly available sequences are provided with associated metadata in Supplementary Table S2.

To assess to what degree the invasive populations of *A. lobifera* differ from the source Indo-Pacific populations*,* we constructed phylogenetic trees and compared the distribution of patristic distances among sequences in the different genetic types of the species as identified by Prazeres et al.^17^. The patristic distances were analyzed for intra-genomic variability (genetic distances among sequences within the same specimen), for population-level variability (genetic distances among sequences from different specimens occurring at the same population), for regional variability (genetic distances among sequences from specimens occurring at different populations within the same oceanic basin) and for geographical variability (genetic distances among sequences from specimens occurring in different oceanic basins). To this end, we constructed two phylogenetic inferences, one including all the sequences of the dataset but covering only the SSU fragment, and one including all the sequences generated in this study and covering the SSU and ITS fragment. For each inference, the sequences were automatically aligned with MAFFT^47^ and a phylogenetic tree was inferred using RAxML-NG^48^ with 100 non-parametric bootstraps and using the substitution model TVM + I + G for the SSU alignment and the TIM2ef + I + G4 for the SSU + ITS alignment that were selected with Modeltest-NG^49,50^. Both trees are provided in Supplementary Fig. S1. After the inference, the patristic distances for the SSU tree and for the SSU + ITS tree were calculated and grouped according to the four categories of comparisons.

To identify the factors affecting intra-genomic distances within the invasive genotype, we used beta dispersion analysis based on Principle Coordinate analysis of square-root transformed patristic distances. We calculated the distance-to-centroid for each specimen for further statistical analyses (ANOVA and Wilcoxon test) to quantify the importance of the factors “oceanic basin” and “status of invasion” in structuring genetic diversity. The patristic distances and the statistical analyses were calculated using the packages *ape*^51^ and *vegan*^52^, respectively, from the software R 4.1.1^53^.

To investigate the phylogeographic relationships among the different populations, haplotype networks were constructed for both the SSU alignment and the newly assembled SSU + ITS alignment. We constructed Median-Joining Networks (MJN)^54^, following an algorithm analogous to that proposed by Excoffier and Smouse^55^ that first constructs Minimum Spanning Trees (MSTs) from a matrix of pairwise distances (absolute number of differences) among haplotypes and includes all possible MSTs using the parsimony criterion to infer and add missing node haplotypes to the MJN graph. We defined ε = 0 for a more stringent distance criterion to select the most parsimonious pathway^56^. To allow the comparison of the population structure within the different genotypes, an analysis of molecular variance (AMOVA) between and within oceanic basins and populations was conducted for each genotype.

In addition to the AMOVA, we also calculated the phi-statistics (Fst) that refers to relative contributions of between-population variations to the overall genetic variation in the whole dataset. The groups were tested for adherence to neutrality (random evolution) assumptions, with Tajima's D^57^. Negative values of Tajima's D indicate an excess of low-frequency polymorphisms, consistent with positive directional selection or recent population expansion, whereas positive values indicate an excess of intermediate frequency polymorphism potentially due to balancing selection or population contraction^58^. Nucleotide diversity, number of haplotypes, and number of segregating sites were also calculated to investigate the degree of polymorphism within the genotypes. The networks and AMOVA were performed in the software PopART 1.7^56^, and the genetic diversity indexes and Tajima’s D were calculated in the packages *haplotypes*^59^, *ape*^51^, and *pegas*^60^, from the software R 4.1.1^53^.

## Results

Our field sampling in 2019 revealed that the invasion front of *A. lobifera* along the eastern coast of Sicily reached at least Plemmirio, but not beyond Brucoli. At the same time, we observed that the abundance of *Amphistegina* in Sicily increased dramatically compared to the observations by Guastella et al.^27^. For instance, in Capo Passero the species represented 35% of the foraminifera in 2017 and in our sampling in 2019 the relative abundance has risen to 75%. In the other two sites where we found *A. lobifera* (Arenella and Plemmirio), the species represented 17 and 49% of the foraminifera (Fig. 1, Supplementary Table S1). Thus, the invasive population has now become a major component of the assemblages on the southern coast of Sicily, but the invasion seems to have halted at the 14 °C winter isotherm along the northern Sicilian coast, which is considered to represent the thermal limit for the species^12^. This means that the Sicily populations sampled for genetic analyses (Fig. 1) represent not only the invasion front but also an established invasive population in the South of the sampled coastal transect.

For the analysis of genetic variability of the invasive and source populations, we successfully amplified the SSU + ITS of four to 11 specimens per locality and sequenced between two to 20 clones per specimen, resulting in 373 SSU + ITS sequences from 37 specimens. For the SSU analysis, we combined the 259 sequences acquired from NCBI^17^ with the new data, resulting in a dataset with 632 SSU sequences from 88 specimens (Supplementary Table S2). The phylogenetic tree inference conducted on the SSU fragment showed that all the newly sampled specimens represent lineage Ia as defined by Prazeres et al.^17^, confirming that the invasive population is sourced exclusively from the Red Sea, where only that genotype occurs.

**Figure 2:**
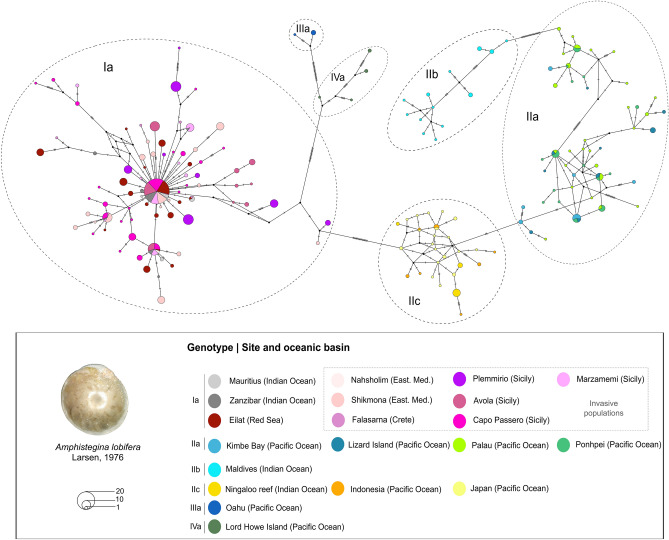
Median-joining network based on SSU rRNA gene sequences of *Amphistegina lobifera* populations, including the invasive genotype Ia and the invasion front in Sicily.

In the median-joining haplotype network, 247 haplotypes and 387 segregating (polymorphic) sites were observed across the 632 sequences in the SSU alignment (Fig. 2). The structure of the network was consistent with the phylogenetic tree with the four lineages being clearly separated. No further structure was observed within genotype Ia, where the invasive Mediterranean populations, the native population in the Red Sea and the Western-Indian Ocean populations share the same common haplotypes and there is no evidence for a systematic (geographical) divergence among them.

The AMOVA of the SSU alignment revealed that within all genotypes of *A. lobifera*, the largest part of the overall genetic variation was explained by variation within populations (specimens collected from the same locality) compared to variation among oceanic basins or among populations, including within the invasive genotype Ia. The genotype Ia showed the lowest nucleotide diversity, indicating a reduced degree of polymorphism and mutation (Table 1) and it was the only genotype that showed significant deviation from neutrality (i.e., evolving randomly) based on Tajima’s D (Tajima’s D = − 2.92, *p* = 0.003). The negative Tajima’s D value indicates fewer haplotypes than segregating sites, which is a sign of an excess of rare alleles and can be considered an indicator of population expansion after a recent bottleneck or recent selective sweep^57,61,62^.

**Table 1:** AMOVA and genetic analyses of *Amphistegina lobifera* genotypes with two or more populations (Ia, IIa and IIc).

Dataset	Alignment	Population	Oceanicbasin	Sequences	AMOVA					Nucleotide diversity	Number haplotypes	Number segregating sites	Tajima's D statistic	Tajima (*p*)
					Variation	Df	Variation (%)	Phi st	Phi st (p) permuted					
SSU	Genotype Ia	Mauritius	West-Ind	11				0.122	**< 0.001**	0.0078	158	252	− **2.92**	**0.003**
		Zanzibar	West-Ind	23	Among oceanic basins	4	− 8.6*							
		Falasarna	Crete	6	Among populations	5	20.8							
		Nahsholim	East-Med	3	Within populations	429	87.8							
		Shikmona	East-Med	72	Total	438								
		Avola	Sicily	61										
		Capo Passero	Sicily	76										
		Plemmirio	Sicily	70										
		Marzamemi	Sicily	33										
		Eilat	Red Sea	84										
		Total		439										
	Genotype IIa	Kimbe-Bay	South-Pacific	19	Among oceanic basins	1	9.9	0.133	**< 0.001**	0.031	68	149	− 1.249	0.211
		Lizard-Island	South-Pacific	19	Among populations	2	3.4							
		Palau	Pacific Ocean	41	Within populations	107	86.7							
		Ponhpei	Pacific Ocean	32	Total	110								
		Total		111										
	Genotype IIc	Ningaloo-reef	Indian Ocean	16	Among oceanic basins	1	40.1	0.433	**< 0.001**	0.025	33	115	− 1.582	0.113
		Indonesia	Pacific Ocean	11	Among populations	1	3.2							
		Japan	Pacific Ocean	24	Within populations	48	56.7							
		Total		51	Total	50								
SSU + ITS	Genotype Ia	Shikmona	East-Med	72	Among oceanic basins	2	− 12.5*	0.135	**< 0.001**	0.019	125	612	− **2.36**	**0.018**
		Avola	Sicily	61	Among populations	2	26.0							
		Capo Passero	Sicily	76	Within populations	356	86.5							
		Plemmirio	Sicily	70	Total	360								
		Eilat	Red Sea	82										
		Total		361										

Significant values are in [bold].

*The negative value in the AMOVA is an artifact of the statistical approach and should be interpreted as 0%, which means that the genetic variation between the oceanic basins does not contribute to the overall total variation.

To assess whether this pattern is due to genetic processes in the invasive populations of the genotype Ia, we carried out the analysis separately for sequences from the Mediterranean and from the Red Sea, and the Indian Ocean (Table 2). This revealed that the deviation from neutrality is not present in the entire genotype, but only in its Red Sea and Mediterranean populations. Finally, we carried out the analysis separately for sequences representing established invaders (Eastern Mediterranean) and the invasion front (Sicily). This revealed that both show deviation from neutrality, and it seems accentuated in the established invaders (Table 2).

**Table 2:** Genetic analyses of invasive genotype Ia constrained by oceanic basins (West-Ind Ocean, Red Sea and Mediterranean Sea) and by different status of invasion in the Mediterranean (established invaders and invasion front).

Dataset	Alignment	Population	Sequences	Nucleotide diversity	Number of haplotypes	Number of segregating sites	Tajima's D statistic	Tajima (p)
SSU	West-Ind Ocean	Mauritius	11	0.024	20	105	− 1.81	0.0702
		Zanzibar	23					
	Red Sea	Eilat	84	0.005	22	279	− **3.38**	**0.0007**
	Med Sea (all)	Falasarna	6	0.010	91	513	− **3.05**	**0.0023**
		Nahsholim	3					
		Shikmona	72					
		Avola	61					
		Capo Passero	76					
		Plemmirio	70					
		Marzamemi	33					
	Med Sea (established invaders)	Falasarna	6	0.007	25	465	− **3.42**	**0.0006**
		Nahsholim	3					
		Shikmona	72					
	Med Sea (invasion front)	Avola	61	0.011	63	202	− **2.62**	**0.0087**
		Capo Passero	76					
		Plemmirio	70					
		Marzamemi	33					
SSU + ITS	Red Sea	Eilat	82	0.018	29	420	− **2.60**	**0.0093**
	Med Sea (all)	Shikmona	72	0.019	97	535	− **2.34**	**0.0193**
		Avola	61					
		Capo Passero	76					
		Plemmirio	70					
	Med Sea (established invaders)	Shikmona	72	0.017	22	365	− **2.60**	**0.0095**
	Med Sea (invasion front)	Avola	61	0.019	72	477	− **2.31**	**0.0202**
		Capo Passero	76					
		Plemmirio	70					

Significant values are in [bold].

**Figure 3:**
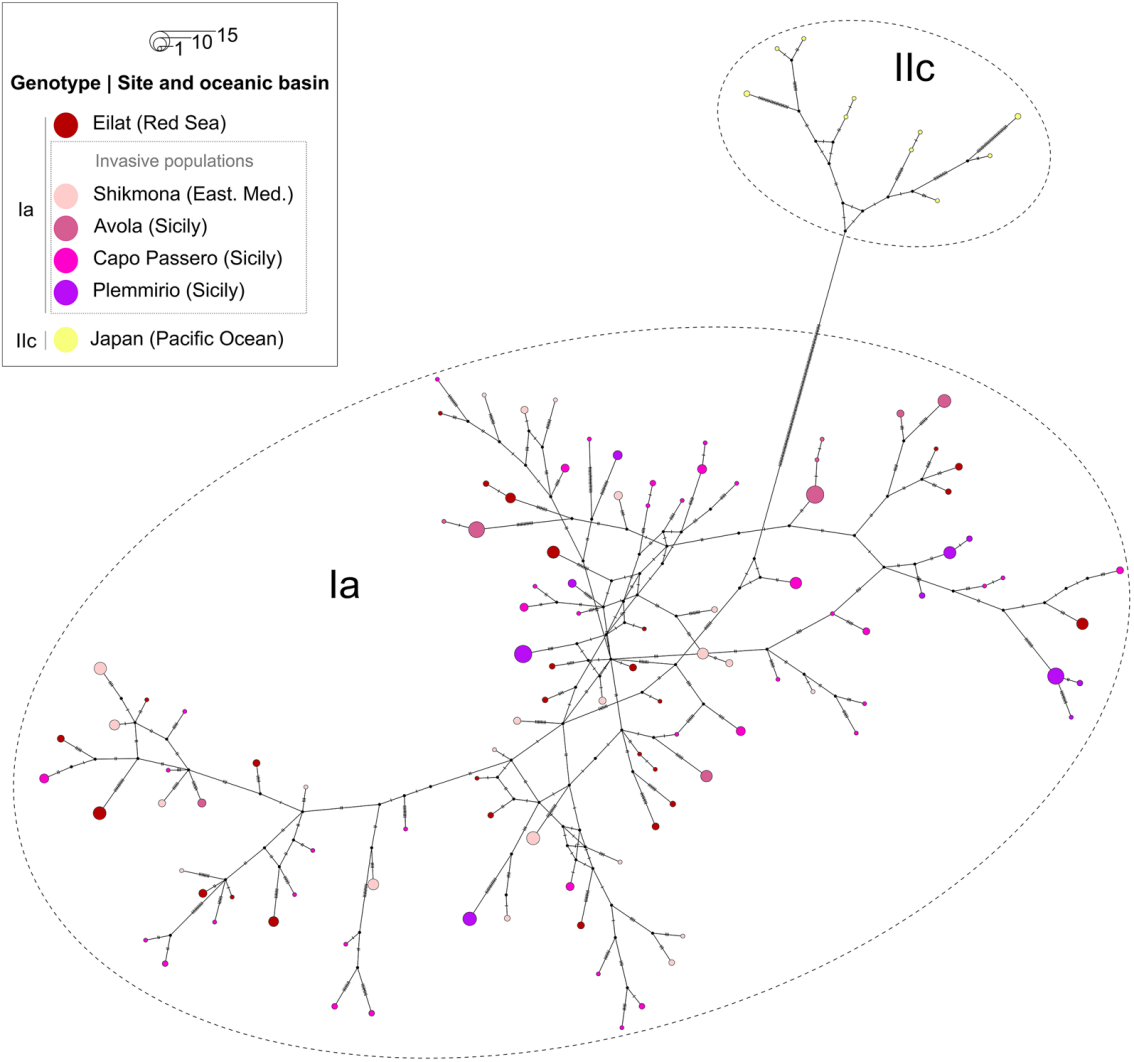
Median-joining network based on SSU + ITS regions of rRNA gene sequences of *Amphistegina lobifera* populations from invasive genotype Ia and out-group (genotype IIc).

As expected, given the known faster mutation rate in the ITS fragment^41^, the SSU + ITS haplotype network showed much higher polymorphism, with 130 haplotypes and 895 segregating sites across 373 sequences (Fig. 3). The network revealed two main haplogroups corresponding to the invasive genotypes Ia and the outgroup genotype IIc from Japan, with neither haplogroup possessing a shared central haplotype.

Like for the SSU alignment, the AMOVA comparing the different oceanic basins within the invasive genotype Ia (Table 1) revealed that the genetic variation is higher within populations (86.5%) than among oceanic basins (− 12.5%) or among populations (26.0%). The negative value in the AMOVA is an artifact of the statistical approach and should be interpreted as 0%, which means that the genetic variation between the oceanic basins does not contribute to the overall total variation at all. This is also shown by the low values of Fst (0.135) and nucleotide diversity (0.019) within genotype Ia (Table 1). Like for the SSU, the Tajima’s D revealed a significant departure from neutrality (Table 1) and indicated population size expansion (e.g., after a bottleneck or a selective sweep) and this pattern is observed in both Red Sea and Mediterranean populations (Table 2). Within the Mediterranean population the deviation from neutrality is observed in both established and new invaders.

**Figure 4:**
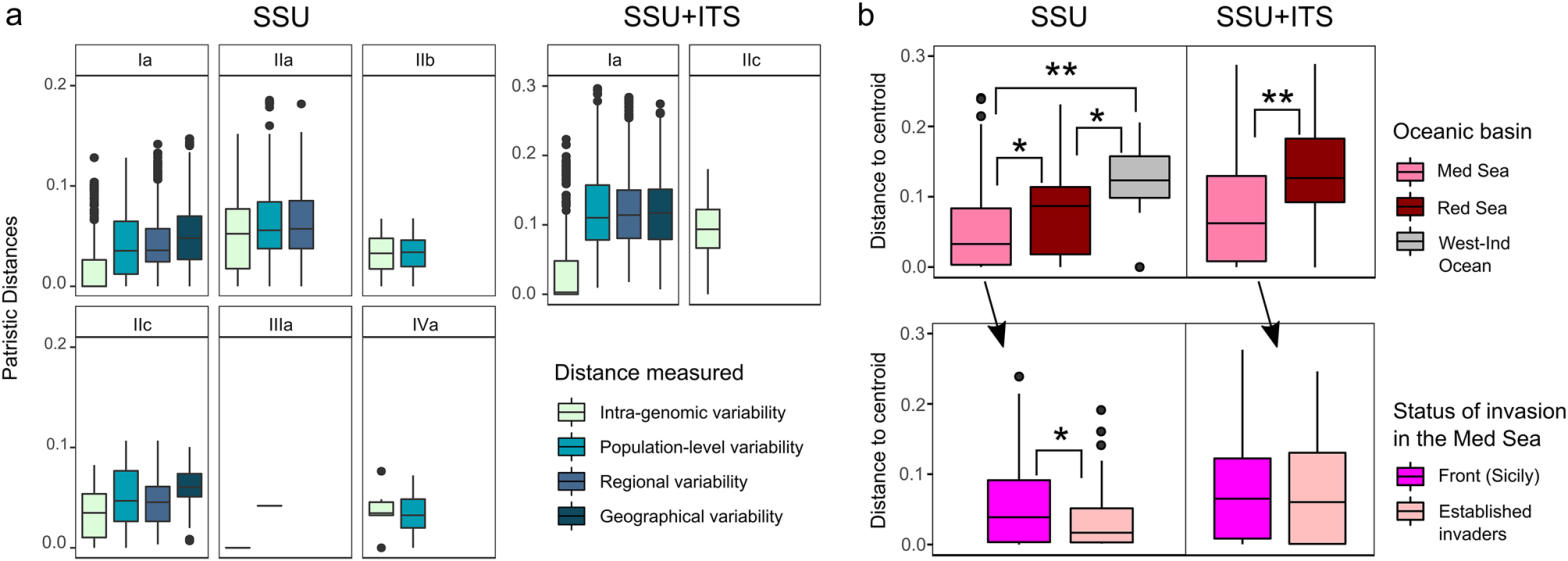
Patristic distances calculated based on phylogenetic trees for the SSU and SSU + ITS rRNA gene sequences of *Amphistegina lobifera* including all genotypes (**a**) and beta dispersion analysis of intra-genomic distances within invasive genotype Ia: by oceanic basin (**b**, top panel) and by status of invasion in the Mediterranean Sea (**b**, bottom panel). Stars represent levels of significance in the Wilcoxon test shown in Table 3.

The lack of geographical structure in the haplotype networks is reflected by the distribution of patristic distances among sequences (Fig. 4). The patristic distances calculated to determine the amount of variability at the intra-genomic level (different clones from same specimen), at the population level (different specimens from same population), at the regional level (different populations from same oceanic basin) and at the geographical level (different oceanic basins) revealed little evidence for an increase in genetic divergence with geographical distance within the invasive genotype Ia. The pattern appeared both in the SSU and SSU + ITS (Fig. 4a) analyses, although in the latter the distances were lower.

The most striking pattern revealed by the patristic distances for both the SSU and the SSU + ITS data is the large reduction of intra-genomic variability in the invasive genotype Ia (Fig. 4a). This observation is highlighted when the invasive genotype Ia is compared to the other genotypes, none of which shows such a large reduction in the intra-genomic variability, except for the genotype IIIa that has too few sequences. To characterize the nature of this apparent reduction in intra-genomic variability, we compared the intra-genomic distance to centroid in the beta dispersion analysis in different populations of the invasive genotype Ia (Fig. 4b and Table 3).

**Table 3:** ANOVA to test for factors affecting intra-genomic variability within invasive genotype (Ia) of *Amphistegina lobifera* and Wilcoxon test for pairwise comparisons of different oceanic basins and different status of invasion.

ANOVA				Wilcoxon pairwise test					
Data	Factor	Df	R2	Comparison	Group 1	Group 2	*p*	p adj	Level of significance
SSU (Ia)	Oceanic basin	2	0.144	Oceanic basins	West-Ind Ocean	Med Sea	1.23E−11	3.69E-11	**
	Population	7	0.119		West-Ind Ocean	Red Sea	3.30E−03	3.30E−03	*
	Specimen	43	0.520		Med Sea	Red Sea	1.42E−04	2.13E−04	*
	Residuals	386	0.218						
SSU (Ia–Med Sea)	Status of invasion	1	0.018	Status of invasion	Med Sea (invasion front)	Med Sea (established invaders)	3.44E−02	3.44E−02	*
	Population	5	0.193						
	Specimen	30	0.564						
	Residuals	284	0.224						
SSU + ITS (Ia)	Oceanic basin	1	0.092	Oceanic basins	Med Sea	Red Sea	2.00E−08	2.00E−08	**
	Population	3	0.109						
	Specimen	31	0.502						
	Residuals	324	0.297						
SSU + ITS (Ia–Med Sea)	Status of invasion	1	0.004	Status of invasion	–	–	–	–	
	Population	2	0.158						
	Specimen	23	0.546						
	Residuals	251	0.292						

* p < 0.05; ** p < 1E−6

This analysis reveals that the large reduction in the intra-genomic distance is present in populations from the Red Sea and significantly accentuated in the Mediterranean, both for the SSU and SSU + ITS (Fig. 4b). The significant reduction in the intra-genomic distances in the Mediterranean is observed both among the established invaders and in populations at the invasion front in Sicily (Fig. 4b). The degree of reduction is similar for all invasive populations with the small differences indicated in the SSU dataset not confirmed by the more informative SSU + ITS analysis (Fig. 4b).

## Discussion

The observed lack of genetic difference between the invasive Mediterranean populations and the source population in the Red Sea (Figs. 2, 3) confirms the postulated large dispersal potential of *A. lobifera*^17^. It also indicates that the invasion must have involved many of the genotypes present in the Red Sea population, rather than a hypothetical pre-adapted subtype. This means that any adaptation facilitating the invasion success was already present in the source population and the opening of the Suez Canal represented an artificial removal of an obstacle for a population that would have otherwise been able to expand beyond the Red Sea. Simultaneously, there does not seem to be any evidence for genetic differentiation of the invasive populations. Theoretically, the time since the invasion may have been too short for unique mutations to accumulate, but this is unlikely considering the observed high within-population variability that demonstrates that the ITS is a suitable marker to capture such a process. Instead, it is more likely that genetic differentiation in the Mediterranean is counteracted by continuous re-seeding by populations from the Red Sea.

At the same time, we observe that the invasive and Red Sea populations show reduced genetic diversity compared to the Indian Ocean populations of the genotype Ia (Table 2). This pattern is consistent with a genetic bottleneck, which is an expected phenomenon associated with invasion (e.g.,^63–65^). Alternatively, the lower sequence diversity could also be a consequence of a selective sweep^61,62^. In this scenario, the reduced diversity in the observed marker could signal strong positive selection against another allele located in the proximity, indicating that the invasion could be associated with the presence of some particularly favorable traits. However, the pattern of low nucleotide diversity and high polymorphism is observed already in the source population from the Red Sea. This would imply that the observed bottleneck or selective sweep already affected the Red Sea population and was not associated with the Lessepsian invasion. Indeed, during each glacial sea level lowstand, the Red Sea becomes hypersaline and inhospitable to most marine organisms, and the basin is repopulated during each deglaciation from the Indian Ocean^66^, with the last such event dating to about 11,000 years ago (e.g.,^67^). The observed reduced genetic diversity could be the result of this last population expansion and seems consistent with a bridgehead effect^68^, where the source population for the invasion into the Mediterranean Sea was in fact a result of a previous expansion from the Indian Ocean into the Red Sea. This effect could have preconditioned the Red Sea population towards a greater invasiveness, thus facilitating the subsequent colonization of the Mediterranean, but further reducing the genetic variability of the invasive population.

This reduced genetic variability observed in the Mediterranean Sea is consistent with a recent invasion. Previous studies suggested that *Amphistegina* may have invaded the Mediterranean Sea on earlier occasions (e.g., pre-historic occurrence during the Pleistocene^69,70^), or even that *A. lobifera* is native to the Mediterranean Sea^71^. The genetic structure of all Mediterranean populations analyzed to date indicate that if *Amphistegina* colonized the Mediterranean Sea in a more distnat past, it must have failed to establish a permanent population, until the most recent invasion event. If the current *A. lobifera* populations occurring in the Mediterranean Sea would be descending from ancient native or earlier Pleistocene invasive populations, then we should have observed distinct genetic differences compared to the Red Sea population, which was not the case.

### Suppression of sexual reproduction in the Mediterranean populations

Surprisingly, rather than a signal of genetic differentiation within the invading populations, we observe a strong and significant reduction of gene-copy variability throughout the Mediterranean populations (Fig. 4; Table 3), which was clearly stronger than in the source population from the Red Sea. This suppression of variability between copy variants in the invasive populations is associated with the retention of high genetic variation among specimens of the same population, requiring an explanation that reduces variability within a genome but not among individuals. One might argue that this low genetic diversity between copy variants observed in Mediterranean populations could be the result of gene surfing, i.e., random loss of genetic diversity over space during range expansion^72^. However, the loss is likely not random, because it affected all the different types invading the Mediterranean. If it would occur in only one population, then we should see reduced intra-genomic variability and reduced variability among individuals as well. Therefore, the most plausible alternative that explains both, the reduced intra-genomic variability and a high genetic variation among specimens of the same population, would be a change in the reproductive strategy towards the suppression of meiotic recombination. This is because sexual reproduction with recombination would be expected to promote genetic variation both among and within individuals. Hence, the observed reduction in intra-genomic variability could be a consequence of a shift towards an asexual reproductive mode that favors gene conversion-like processes leading to a loss of heterozygosity (e.g., as observed in *Daphnia* by Tucker et al.^73^ and in *Trypanosoma* by Weir et al.^74^).

Such gene conversion processes during asexual reproduction could lead to homogenization within a genome. In the presence of continuous re-seeding of the invasive populations from the Red Sea, the large variability among individuals would be preserved. Therefore, we speculate that the genetic structure of the invasive population reflects two processes: high dispersal potential of a pre-adapted Red Sea source population and a suppression of sexual reproduction. It is important to highlight, however, that the observed homogenization could also be a result of concerted evolution, where multiple copies of a gene within a genome evolve in a coordinated manner^75,76^. However, such process should not be acting within the Mediterranean population only, and it is therefore less likely that this process would be responsible for the particular profile we observed.

Like many other foraminifera *Amphistegina* is known to have a trimorphic life cycle involving a sexual generation (agamont) and two asexual generations (gamont and schizont), with no necessity for strict alternation between asexual and sexual generations (e.g.,^77–83^). In symbiotic organisms, a change in reproduction strategy can also be linked to the process of obtaining and maintaining symbiosis. Like in many other symbiont-bearing organisms, in LBFs, asexual reproduction is associated with a vertical transfer of symbionts to the offspring. Offspring generated by multiple fission has a large size (~ 40–50 µm) and can receive the cytoplasm of the parent together with the symbionts^78^. On the other hand, the tiny gametes (2–3 µm) cannot carry the symbionts and therefore the zygotes must acquire them from the environment. As a result, in cyclic schizogony (i.e., no alternation with sexual reproduction), the symbiont culture is maintained without the need to receive new symbionts from the environment^78^, although such uptake of symbionts from the environment (horizontal transmission) remains a possibility. This could provide an explanation for the lack of sexual reproduction in the invasive *A. lobifera*. In this scenario, the ability to reproduce by cyclic schizogony would represent an advantage, or even a prerequisite, for populating regions with environmental conditions that do not allow the acquisition of new symbionts^77^. However, it is unclear whether enhanced cyclic schizogony in the Mediterranean individuals is an actual adaptation or a plastic response from *A. lobifera* facing extreme conditions. Therefore, it is equally possible that the invaders do not engage in sexual reproduction (gamete release), or they do, and their attempts are not successful.

Interestingly, the slightly but significantly reduced intra-genomic variability in the Red Sea population indicates that the same process, but to a lesser degree, may act already on the northern Red Sea populations of the species. Compared to the Indian Ocean range of the species, the northern Red Sea already represents thermal conditions close to the cold limit of the species range. However, the Red-Sea populations possess tolerance to high temperatures^25^. Like in northern Red Sea corals^8^, the retention of high tolerance in *A. lobifera* is likely the result of thermal filtering of Indian Ocean populations entering the Red Sea from the south. Therefore, it is possible that already the northern Red Sea populations of *A. lobifera* live under stressful environmental conditions that lead to partial suppression of sexual reproduction.

There are numerous observations of shifts in reproductive strategy in association with biological invasions and several well-known invasive species are asexual, with most examples known among plants (e.g.,^84–87^). For instance, the reed canary grass *Phalaris arundinacea* (Poaceae) rapid invasion in wetlands in North America has been attributed to clonal subsidy, morphological plasticity, and nutrient availability^88^. And the invasiveness of the aquatic plant *Myriophyllum aquaticum* (Haloragaceae) is regarded to be facilitated by clonal integration, which has been found to significantly improve the growth and photosynthetic performance of daughter ramets^89^.

That marine invasion may impede sexual reproduction among protists has, however, not been documented before. This is significant because sex has an obvious long-term advantage by creating new combinations of genes that allow adaptation to future changing conditions^90^. In contrast, asexual reproduction can be an effective strategy to rapidly increase population size during the colonization of new areas^80^ but has the disadvantage of decreasing the ability of the invasive population to react to future change by adaptation. Therefore, if the invasive *Amphistegina* is unable to reproduce sexually in the Mediterranean, its future proliferation may be affected by the loss of adaptive potential in the face of the projected continued environmental changes in their newly conquered space^91^. Finally, the hypothesis of the loss of sexual reproduction could be tested on other invasive foraminiferal species in the Mediterranean. The outcomes of this analysis may enhance our understanding of adaptive behavioral patterns within this group under diverse stressful conditions and contribute to predicting the species that are more likely to be successful invaders.

## Conclusion

Our results revealed that the invasion of the symbiont-bearing foraminifera *A. lobifera* in the Mediterranean Sea is facilitated by the combination of preadaptation and a high dispersal ability, with sustained re-seeding of the Mediterranean from the Red Sea. The invasion involves many of the genotypes present in the Red Sea population, rather than a specific subtype, indicating that the preadaptation to invasion was widespread in the source population. At the same time, the invasive populations show reduced intragenomic variability associated with sustained high genetic variation among specimens, which can be explained by a lower average heterozygosity due to increased gene conversion during asexual reproduction. The invasion therefore appears to be associated with a sustained change in reproductive strategy towards the abandonment of sex. Either the sexual reproduction is not triggered or cannot be completed due to adverse environmental conditions in the new habitat or, alternatively, because the zygotes have difficulty in acquiring symbionts from the environment. Either way, this discovery provides a new perspective on the cost of invasion in marine protists. If the invasion is facilitated by or requires a shift towards cyclic schizogony, the short-term gain of invasion into new habitats may be offset by a long-term loss of adaptive potential.

### Supplementary Information

Supplementary Information.

Supplementary Table 1.

Supplementary Table 2.

## Data Availability

The dataset with sequences and associated metadata generated during the current study are available in Supplementary Table S2 and in the NCBI repository, under the accession numbers OP610171-OP610543.
