# Supplementary material for: Invasion success of a Lessepsian symbiont-bearing foraminifera linked to high dispersal ability, preadaptation and suppression of sexual reproduction
Source: Sci Rep. 2023 Aug 3;13:12578. doi: 10.1038/s41598-023-39652-y (PMC10400638; doi:10.1038/s41598-023-39652-y)
Supplement: Supplementary file 1 — Supplementary Information. [file 41598_2023_39652_MOESM1_ESM.pdf]

Supplementary information for:

Invasion success in a Lessepsian symbiont-bearing foraminifera linked to high dispersal ability, preadaptation and suppression of sexual reproduction

Débora S. Raposo*[1], Rebecca A. Zufall[2], Antonio Caruso[3], Danna Titelboim[4], Sigal Abramovich[5], Christiane Hassenrück[1,6], Michal Kucera[1], Raphaël Morard[1]

[1] MARUM, Center for Marine Environmental Sciences, Universität Bremen, Bremen, Germany

[2] Department of Biology and Biochemistry, University of Houston, Houston, United States

[3] Dipartimento di Scienze della Terra e del Mare, Università degli studi di Palermo, Palermo, Italy

[4] School of Earth Science, University of Bristol, Bristol, United Kingdom

[5] Department of Earth and Environmental Sciences, Ben Gurion University of the Negev, Beer Sheva, Israel

[6] Department of Biological Oceanography, Leibniz Institute for Baltic Sea Research Warnemünde (IOW), Rostock-Warnemünde, Germany

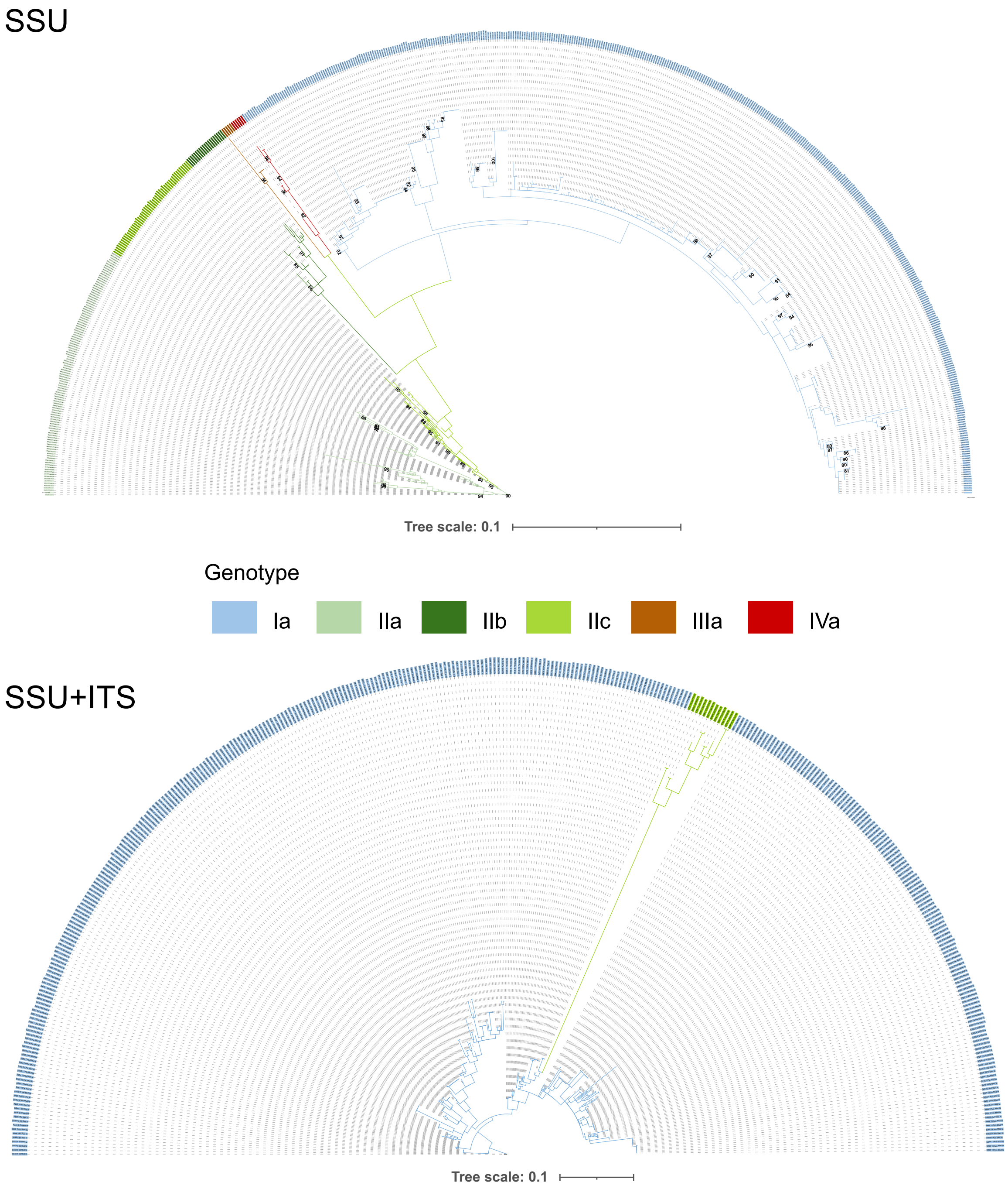

Figure S1. Phylogenetic trees based on the SSU and the SSU+IST regions of rRNA gene sequences of *Amphistegina lobifera* populations for all genotypes, including the invasive genotype Ia.
